# Signalling mechanisms driving homeostatic and inflammatory effects of interleukin-15 on tissue lymphocytes

**DOI:** 10.1093/discim/kyae002

**Published:** 2024-01-30

**Authors:** Neema Skariah, Olivia J James, Mahima Swamy

**Affiliations:** MRC Protein Phosphorylation and Ubiquitylation Unit, School of Life Sciences, University of Dundee, Dundee DD1 5EH, UK; MRC Protein Phosphorylation and Ubiquitylation Unit, School of Life Sciences, University of Dundee, Dundee DD1 5EH, UK; MRC Protein Phosphorylation and Ubiquitylation Unit, School of Life Sciences, University of Dundee, Dundee DD1 5EH, UK

**Keywords:** interleukin-15, innate-like T cells, natural killer cells, signalling, autoimmunity

## Abstract

There is an intriguing dichotomy in the function of cytokine interleukin-15—at low levels, it is required for the homeostasis of the immune system, yet when it is upregulated in response to pathogenic infections or in autoimmunity, IL-15 drives inflammation. IL-15 associates with the IL-15Rα within both myeloid and non-haematopoietic cells, where IL-15Rα trans-presents IL-15 in a membrane-bound form to neighboring cells. Alongside homeostatic maintenance of select lymphocyte populations such as NK cells and tissue-resident T cells, when upregulated, IL-15 also promotes inflammatory outcomes by driving effector function and cytotoxicity in NK cells and T cells. As chronic over-expression of IL-15 can lead to autoimmunity, IL-15 expression is tightly regulated. Thus, blocking dysregulated IL-15 and its downstream signalling pathways are avenues for immunotherapy. In this review we discuss the molecular pathways involved in IL-15 signalling and how these pathways contribute to both homeostatic and inflammatory functions in IL-15-dependent mature lymphoid populations, focusing on innate, and innate-like lymphocytes in tissues.

## Introduction

Immune cells work in a coordinated manner to defend the body against various pathogenic insults. Cytokines act as soluble immune communicators that coordinate responses between different immune cells. While cytokines are essential for mounting a successful immune response against infections, dysregulated expression of cytokines can damage surrounding tissues. Interleukin (IL)-15 is an intriguing cytokine for many reasons—unlike other cytokines, it is mostly trans-presented in a membrane bound form to its target cells, enabling very short-range communication, second, it is produced not just by immune cells, but also by stromal cells, particularly in mucosal tissues, where it can act as a danger signal, and, while it maintains immune cell homeostasis at low levels, at high levels or prolonged up-regulation it causes pathogenesis and autoimmunity [1].

**Figure 1:**
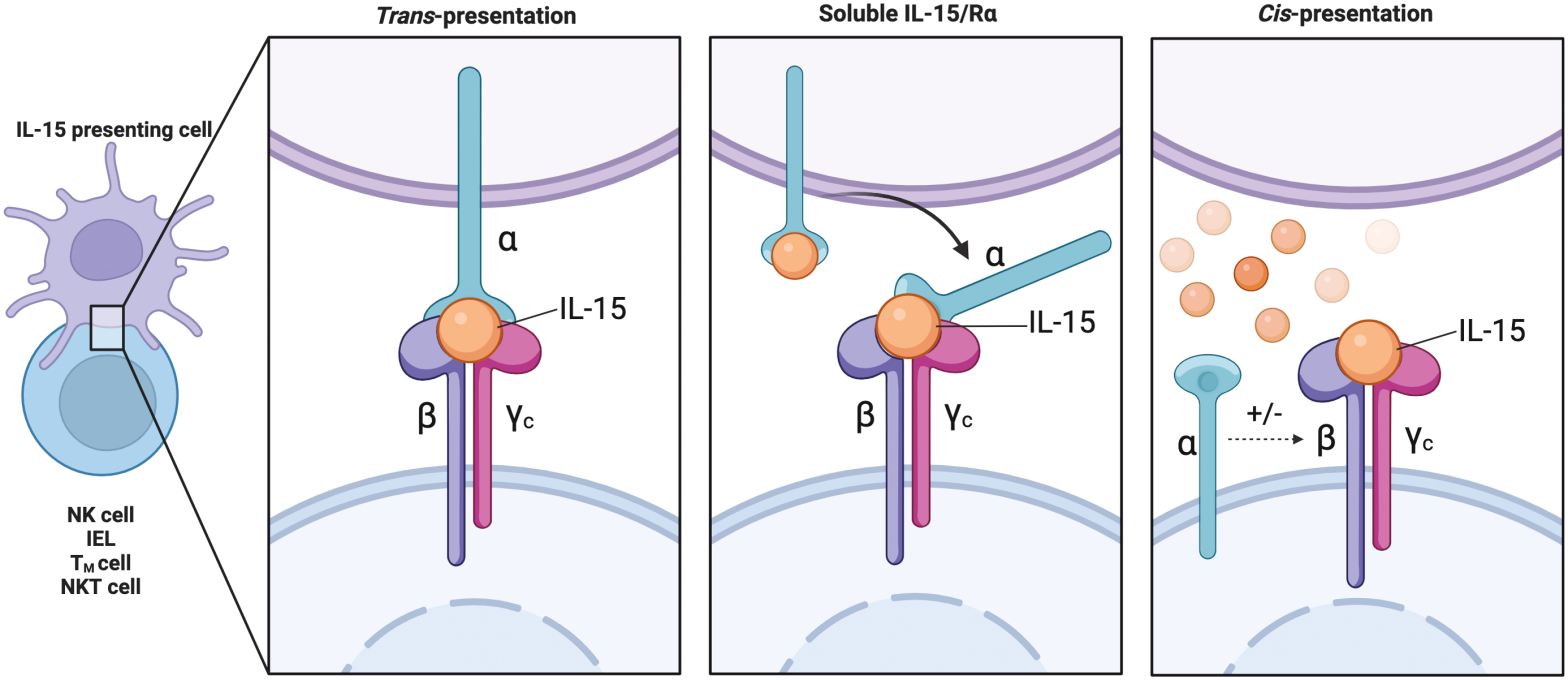
Modes of IL-15 presentation. IL-15 functions predominantly by trans-presentation where membrane bound IL-15/IL-Rα is presented by IL-15 presenting cells to the heterodimeric IL-2Rβ/γc present on lymphocytes. Alternatively, soluble IL-15/IL-15Rα shed by IL-15 presenting cells can also bind to heterodimeric receptors on lymphocytes to activate signalling. In addition to these mechanisms, binding of soluble IL-15 to IL-15Rα can allow signalling of adjacent IL-2Rβ/γc present on the same cell through cis-presentation. Created in Biorender.com.

IL-15 is a 14-15 kDa protein belonging to the common γ-chain (γc) family of cytokines, that includes IL-2, IL-4, IL-7, IL-9, and IL-21 [2]. IL-15 has diverse effects on various immune cells such as lymphocytes, mast cells, monocytes, macrophages, neutrophils, and eosinophils [3–10]. In lymphocytes, IL-15 signals through a hetero-trimeric receptor consisting of 3 subunits: a γ chain (γc/CD132) that it shares with the other members of the family; a β chain (CD122/IL-2Rβ) that it shares with IL-2, and a private α chain (IL-15Rα/CD215). IL-15 on its own binds with low/intermediate affinity to heterodimeric IL-2Rβ/γc receptor and with high affinity to its hetero-trimeric IL-15Rα/IL-2Rβ/γc receptor [11]. Binding of IL-15 to IL-15Rα enhances the binding affinity of the cytokine to IL-2Rβ/γc. IL-15 signals can be transmitted by trans-presentation or cis-presentation of IL-15 to its receptor (Fig. 1) [12]. However, trans-presentation appears to be the main mechanism driving IL-15 biology, as soluble IL-15 is only found complexed to IL-15Rα in both human and mouse serum [13].

Despite sharing the heterodimeric IL-2Rβ/γc receptor subunits with IL-2, IL-2, and IL-15 have distinct roles *in vivo*, suggesting that α chain receptor plays an especially significant role in the specificity of action [14]. The most prominent difference is that IL-2 is a secreted diffusible molecule that can bind to the hetero-trimeric IL-2 receptor on lymphocytes, whereas IL-15 is mainly found as a membrane-bound complex with IL-15Rα. In tissues, IL-15 signalling is induced by the trans-presentation of the IL15/IL-15Rα complex to the heterodimeric IL-2Rβγ receptor on lymphocytes [15]. While IL-2 is required for T-cell expansion and maintenance of regulatory T cells (T_reg_), IL-15 is essential for the development and maintenance of natural killer (NK) cells, NK like T cells (NKT), CD8^+^ memory T (T_MEM_), innate-like lymphoid cells (ILCs), dendritic epidermal γδ T cells (DETC), and intestinal intraepithelial T lymphocytes (IEL) [16–22]. Further, while IL-2 promotes activation-induced cell death and tolerance in many of these cell types, IL-15 is known to prolong the survival of cytotoxic lineages and promote autoimmunity [14]. In tissues, IL-15 is upregulated in infection and stress, and can drive the cytotoxic effector functions of tissue-resident T cells, a key function for protecting the tissue against pathogens [1]. Here, we discuss the regulation of IL-15 expression and delineate the homeostatic and proinflammatory signalling of IL-15 in lymphocytes and their role in driving the pathogenesis of several autoimmune conditions.

### Regulation of IL-15 expression

**Figure 2:**
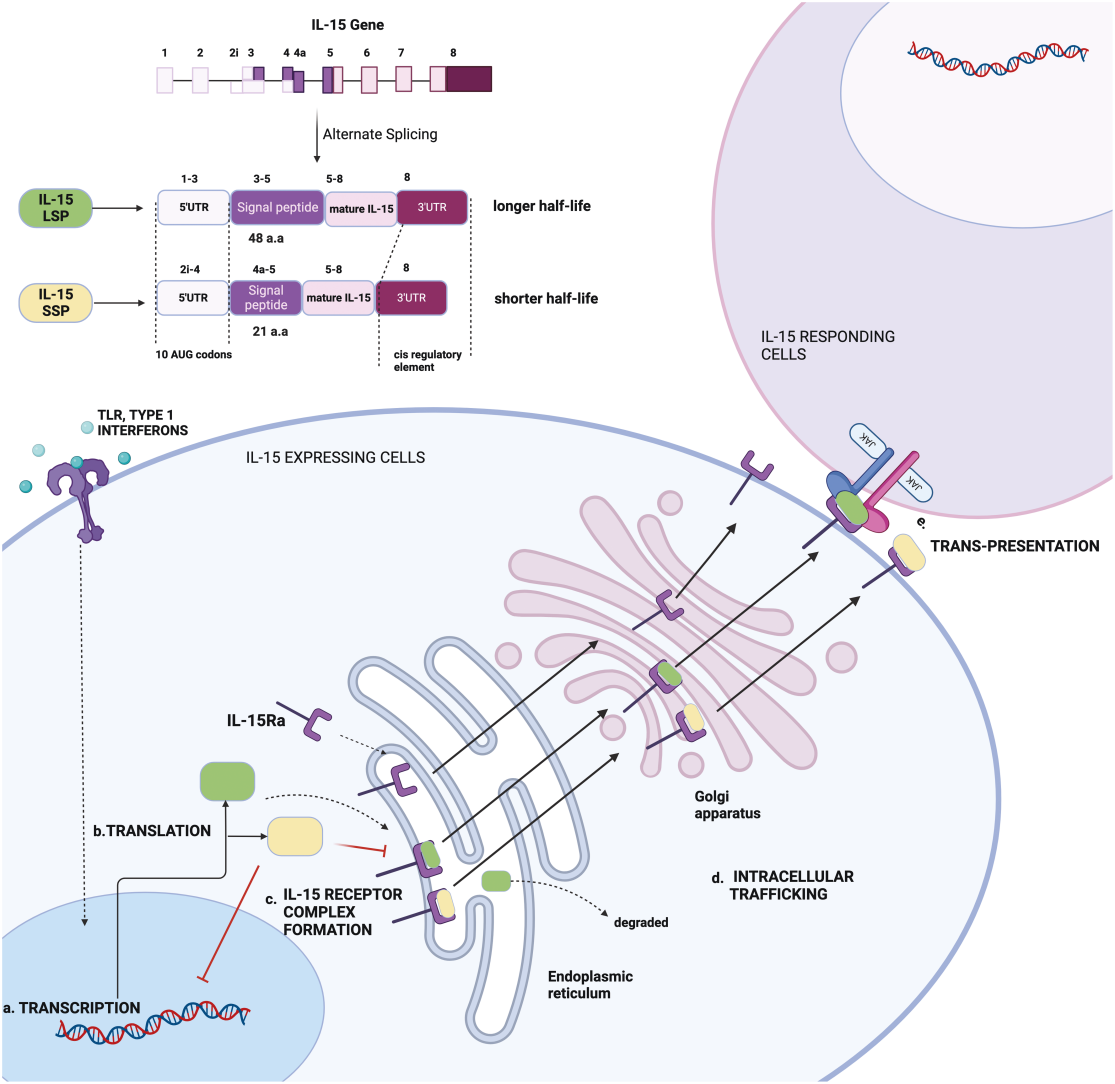
Multifaceted regulation of IL-15 expression. IL-15 expression is modulated at various levels, such as transcription, translation, receptor complex formation, intracellular trafficking, and surface presentation. IL-15 transcription is induced in IL-15-expressing cells by Toll-like receptor or type I interferon receptor activation. Alternate splicing of Il15 results in the formation of two isoforms of IL-15 containing a short (21 amino acids) signal peptide (IL-15SSP) and long (48 amino acids) signal peptide (IL-15LSP) where IL-15SSP inhibits the transcription of IL-15LSP. Translational efficiency is reduced due to 10AUG codons in the 5ʹUTR and a negative cis-regulatory element in the C terminal. In the cell, IL-15 forms a complex with IL-15Rα in the endoplasmic reticulum. IL-15SSP competes with IL-15LSP for binding to IL-15Rα. IL-15Rα/IL-15 complex is stable, escapes degradation, moves through the Golgi complex, and is expressed on the cell’s surface, where it is trans-presented to the IL-15 responding cells. Created in Biorender.com.

The IL15 gene is mapped on chromosome 4q31 in humans and the central region of chromosome 8 in mouse [23]. *Il15* mRNA has been detected in various tissues, including the placenta, skeletal muscle, kidney, lung, heart, fibroblasts, epithelial cells, and monocytes [24, 25], but the protein is mainly expressed by macrophages, dendritic cells, epithelial cells, and activated fibroblasts, suggesting tight regulation of its expression [26–28]. Regulation of IL-15 expression is seen at various levels of protein synthesis with modest transcriptional and predominant post-transcriptional regulation, which explains the disconnect between the widespread mRNA expression and little to no protein expression [23]. Studies using IL-15 reporter mice showed that IL-15 promoter activity was differentially regulated, and its expression was limited to myeloid lineages and tissue-specific epithelial cells [28]. Toll-like receptor ligands and type I interferons can induce the transcription of IL-15 in dendritic cells [29, 30]. Translation of IL-15 is hindered by the presence of 10 AUG sequences in the 5ʹ UTR of human IL-15, its unusual signal peptides (SP), and a negative regulatory element at the C-terminus of the IL-15 mature protein coding sequence (Fig. 2) [25, 31, 32]. Alternative splicing into two forms with different lengths of signal peptides further contributes to the regulation of IL-15 expression [32]. In COS cells transfected with the long signal peptide (LSP) construct, IL-15 is trafficked more slowly through the cell once it enters the secretory pathway compared to IL-2. However, this was shown to be only partially dependent on the SP [32]. Interestingly, the short signal peptide (SSP) form can block transcription of the LSP form. On the other hand, IL-15 LSP is more stable when complexed with IL-15Rα and can be presented for longer. IL-15Rα bound to IL-15 SSP is degraded more quickly, reducing the bioavailability of IL-15 (Fig. 2) [33]. It is presently unknown if a shift in these regulatory mechanisms underlies the pathogenic manifestations of IL-15, however, it is evident that several mechanisms ensure that IL-15 expression and trans-presentation are tightly regulated.

### IL-15 signal transduction

**Figure 3:**
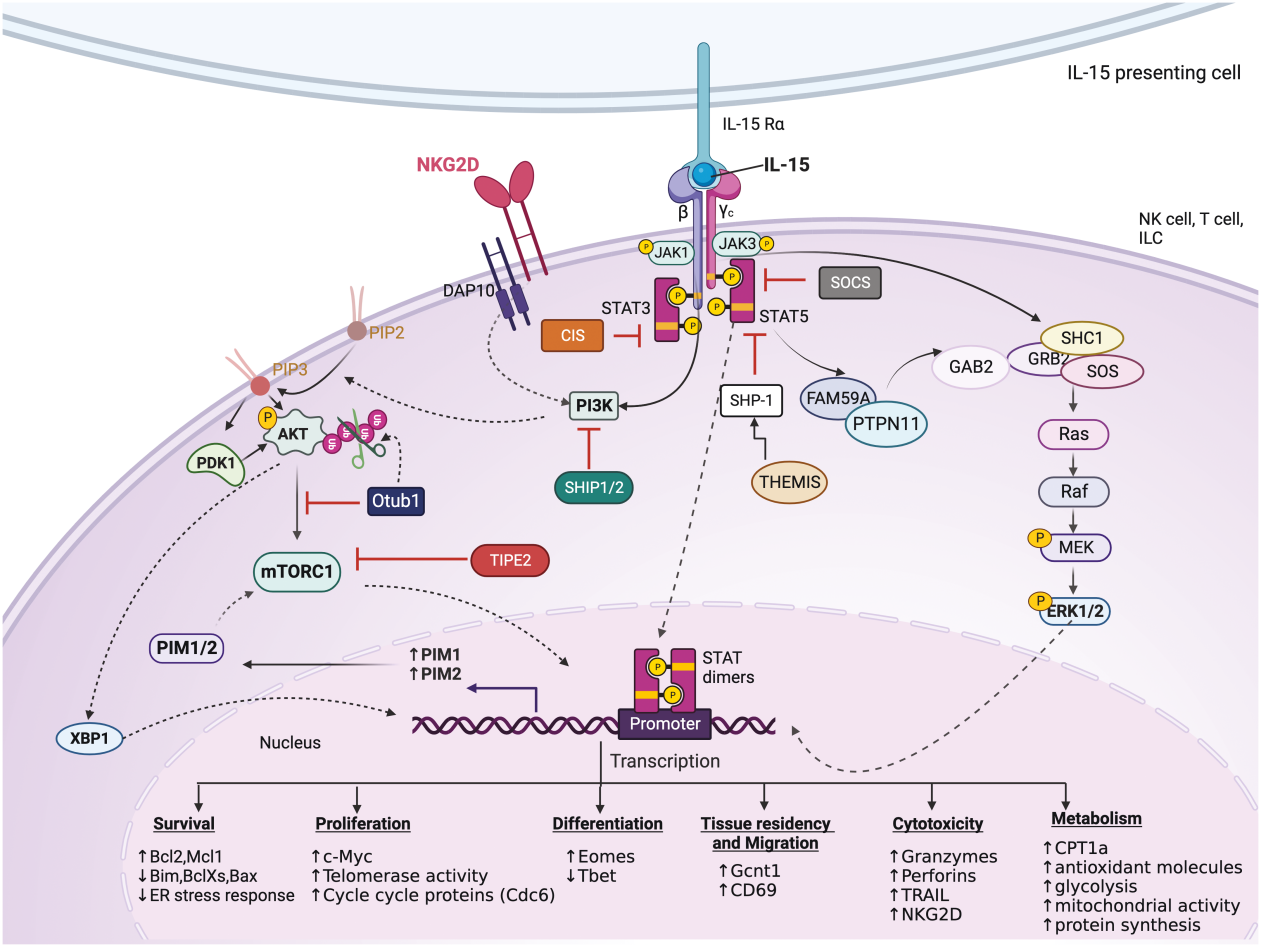
IL-15 signalling in immune cells. Upon IL-15 trans-presentation to heterodimeric IL-2Rβ/γc on the target cell, three main signals are initiated: JAK/STAT pathway, PI3K/AKT/mTOR pathway, and MAPK pathway, to promote various cellular functions such as survival, proliferation, differentiation, motility, metabolism, and cytotoxicity. Activation of the JAK/STAT pathway leads to STAT3/5 dimerization, and STAT dimers translocate to the nucleus to initiate transcription of various genes. Simultaneously, the recruitment of PI3K to the receptor increases PIP_3_, which can activate PDK1 and AKT and initiate further downstream signalling through mTOR. In addition, AKT activation leads to stabilization of XBP1 which can translocate to the nucleus to initiate further transcriptional programs. Meanwhile, the recruitment of various adaptor proteins such as FAM59, PTPN11, GAB2, GRB2, SHC1, and SOS to the activated receptor stimulates the MAP kinase pathway. IL-15 receptor stimulation also activates negative feedback through the upregulation of various negative regulators of the JAK/STAT signalling, such as the SOCS family proteins (SOCS1, CIS, SHP1) and inhibitors of the AKT pathway such as Otub1 and TIPE2. Created in Biorender.com.

Three main signalling pathways are known to be triggered by the engagement of membrane-bound IL-15/Rα with IL-15-receptor βγ chains in responding cells—Janus-associated kinases (JAK)/signal transducers and activator of transcription (STAT), phosphatidylinositol 3 kinases (PI3K)-protein kinase B (AKT), and mitogen-activated protein kinase (MAPK) pathways (Fig. 3) [34]. Receptor engagement activates ubiquitously expressed JAK1 and haematopoietic lineage-specific JAK3 [35]. JAK1/3 phosphorylate specific tyrosine residues that are docking sites for proteins containing Src-homology 2 (SH2) or phospho-tyrosine (pY) binding domains. Phosphorylation of Y536 on the C-terminal tail of IL-2Rβ acts as an anchoring site for the STAT proteins STAT5 and STAT3, which are phosphorylated by JAK kinases [35, 36]. Phosphorylated STATs are released from the receptor, dimerize, translocate to the nucleus, and bind to promoters of target genes involved in cell survival and proliferation (Fig. 3) [34, 37].

The MAPK pathway is simultaneously activated following stimulation with IL-15. Phospho-tyrosine site (Y388) on the IL-2Rβ chain of the active receptor serves as the docking site for pY domains of adaptor protein SHC1. Upon recruitment, SHC1 is phosphorylated and recruits Growth factor receptor-bound protein 2 (GRB2) through its SH2 domains. Through its SH3 domain, GRB2 interacts with the Ras guanine nucleotide exchange factor, Son of Sevenless (SOS), inducing the Ras-Raf-MAPK pathway [36]. Alternatively, IL-15 also induces phosphorylation of the adaptor protein, GRB2-associated and regulator of MAPK protein (GARE1, also known as FAM59A) that interacts with tyrosine–protein phosphatase non-receptor type 11 (PTPN11), which might activate the MAPK pathway through the formation of a complex with GRB2-associated binding protein 2 (GAB2) and its constitutive partner GRB2 [36]. The products of MAPK signalling promote cellular proliferation, cytokine production, and antigen-specific expansion in immune cells (Fig. 3) [34].

Another vital signalling pathway triggered by IL-15 stimulation is the PI3K-AKT pathway. PI3K is recruited and binds to the activated receptor near its lipid targets on the plasma membrane after stimulation. Activated PI3K increases phosphatidylinositol-3,4,5-triphosphate (PI(3,4,5)P_3_) levels, which interact with PDK1 and AKT, leading to their recruitment to the plasma membrane. Interaction between PI(3,4,5)P_3_ and AKT initiates conformational changes in AKT, allowing phosphoinositide-dependent kinase 1(PDK1) phosphorylation of AKT at Thr308 resulting in its activation and further downstream signalling [36, 38]. The PI3K-AKT pathway is particularly important in IL-15-mediated development, expansion, metabolic regulation, and cytotoxic functions (Fig. 3) [39].

### IL-15 signalling pathways driving immune homeostasis

**Figure 4:**
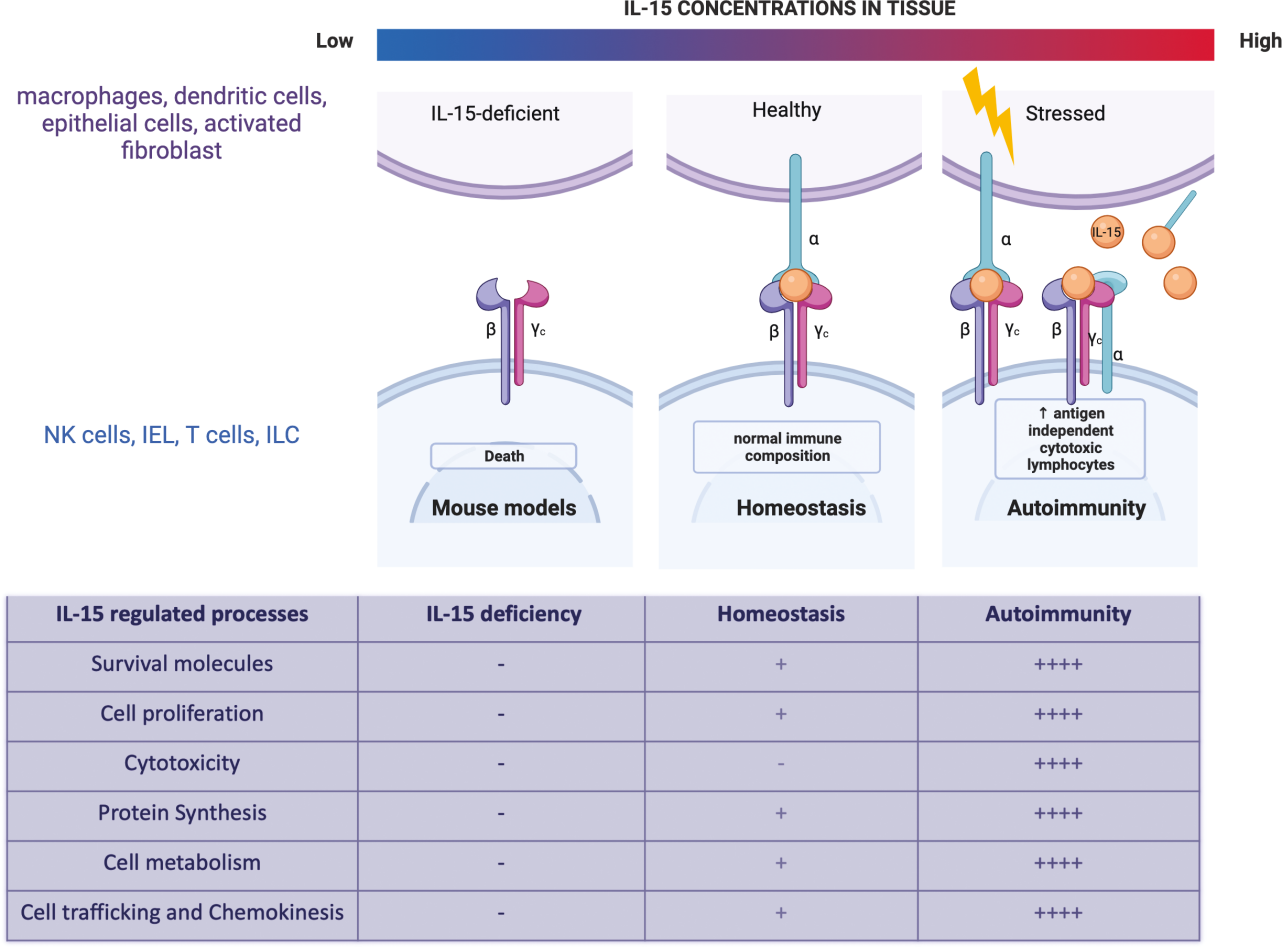
IL-15 as a homeostatic and proinflammatory cytokine. Loss of IL-15 signalling leads to deficiency in immune cell populations such as NK cells, memory T cells, and IELs. IL-15, at moderate levels during steady state, is required to develop and maintain several immune cells such as NK cells, IEL, T_MEM,_ and ILC. At physiological levels, IL-15 regulates apoptosis, proliferation, metabolism, and cell trafficking. However, with an increase in IL-15 expression observed during inflammation and autoimmunity, a robust continuous signalling by IL-15 leads to increase in cytotoxicity in addition to survival, proliferation, metabolism, and cell motility which leads to an imbalance in immune response causing tissue damage.

IL-15 is crucial for the homeostasis of many innate-like lymphocytes. The complementary strategies of either ablation or transgenic expression of IL-15 have highlighted the importance of IL-15 [40, 41]. Genetic ablation of IL-15 caused lymphopenia due to substantially reduced numbers of T_MEM_, NK cells, NKT cells, and IEL in the skin and gut. Conversely, transgenic expression of human IL-15 led to the elevation of these subsets, an effect that could be reversed using an IL-2Rβ blocking antibody that hindered IL-15 activity [42, 43]. IL-15 maintains homeostasis by regulating various mechanisms such as cell survival, proliferation, differentiation, and migration (Figs. 3 and 4).

#### Regulation of lymphocyte survival at steady state

IL-15 promotes cell survival during development or in lymphopenia through JAK/STAT and PI3K/AKT pathway-mediated regulation of both anti- and pro-apoptotic factors of the Bcl-2 protein family. However, IL-15-induced survival mediators vary according to species and cell type. Although IL-15 induces anti-apoptotic protein Bcl2 in multiple cell types and the overexpression of Bcl-2 ultimately rescued NKT cell numbers in IL15^−/−^ mice, it only partially recovered the NK compartment in IL15^−/−^ mice [20, 21, 44, 45]. Along similar lines, IL-15 was able to promote the survival of murine NK cells even in the presence of BH3 mimetic compound ABT 737, which blocks Bcl2 and Bcl-xl [46]. Mechanistically, this was shown to be through the maintenance of expression of anti-apoptotic protein Mcl-1 and reduction of pro-apoptotic protein Bim in NK cells. Bim was reduced through proteasomal degradation induced by IL-15-mediated ERK1/2 phosphorylation of Bim and through transcriptional repression mediated by phosphorylation of Foxo3a by P13K-AKT [46]. Conversely, in human NK cells, IL-15 was shown to maintain Bcl2 expression and reduce Bid abundance without affecting Bcl-xl, Mcl-1, and other BH3 molecules [21, 46–48]. T effector cells also showed a reduction in pro-apoptotic proteins Bcl-Xs and Bax in response to IL-15 in a lymphopenia mouse model [48]. IL-15-induced ERK1/2 dependent phosphorylation of Ser65 of pro-apoptotic Bim(EL) in murine IEL resulted in dissociation of the Bcl2-Bim complex, thus shifting the balance towards pro-survival [49]. In addition to regulation by Bcl2 family proteins, IL-15 could regulate homeostatic cell survival by modulating several ER stress response proteins through an unknown mechanism, thus inhibiting survival defects induced by intense ER stress and PKR-like ER kinase (PERK) expression [50].

#### Cell proliferation at steady state

In addition to survival, antigen-independent homeostatic proliferation induced during lymphopenia restores the lymphocyte pool through homeostatic cytokines such as IL-15. IL-15 controls the expansion of human and murine NK cells, T_MEM_, and γδ T cells [51–54]. JAK-STAT and PI3K-AKT-mTOR pathways were primarily responsible for homeostatic expansion [55]. The transcription factor c-Myc has been implicated downstream of these pathways as a mediator of homeostatic proliferation with T_MEM_ and NK cells showing defective homeostatic proliferation in c-Myc^+/−^ mice [52, 55]. Although c-Myc deficiency led to reduced proliferation in CD8αα IEL, BrdU incorporation studies with Villin/IL-15Rα transgenic mice indicated no difference in IEL proliferation in the presence of overexpressed IL-15 compared to wild-type IEL [56, 57]. The idea that IL-15 drives the proliferation of IEL *in vivo* is conflicted, with many hypothesizing that IEL accumulation in the epithelium during tissue stress or disease is due to an increase in IL-15-mediated survival of IEL [58]. Indeed, overexpression of transgenic Bcl2 in c-Myc deficient mice rescued CD8αα IEL, suggesting that c-Myc, through modulation of Bcl2, predominantly regulates IL-15 mediated survival rather than proliferation [56]. Thus, role of IL-15-driven proliferation at a steady state remains debatable due to lack of mechanistic studies.

#### Differentiation and maintenance of tissue-resident lymphocyte populations at steady state

Maintaining the composition of different immune cell populations is another critical aspect of homeostasis. In addition to survival and homeostatic proliferation, IL-15 maintains the composition of immune cells by regulating various developmental and differentiation/maturation signals in NK cells, NKT cells, CD8 T cells, ILC, DETC, and IEL [19, 20, 59–61]. Expression of nuclear factor NFIL3, a critical regulator of NK cell lineage commitment, is thought to be regulated by IL-15 through PDK1-mTOR signalling [62]. Mechanistically, IL-15 signalling leads to upregulation of mTOR complex 1 (mTORC1) and Tsc1 expression, which prevents exhaustive proliferation of NK cell precursors and thus defective NK cell maturation [63]. In NKT cells, IL-15 regulates *Tbx21* (T-bet) expression, which is essential for their differentiation and upregulation of cytotoxic gene program, including *Ifng* (Interferon γ), *Gzma* (granzyme A), *Gzmc, Hopx* (homeobox protein), and several NK cell receptor genes (*Klra3* [NKG2E], *Klrb1c* [NK1.1], *Klrc1* [NKG2A/B], and *Klrk1* [NKG2D]) [20, 64, 65]. As opposed to NKT cells, IL-15 promoted T_MEM_ by inhibiting T-bet and promoting Eomes upregulation and memory T cell metabolism through the ULK1/Atg7 autophagy pathway in mouse models of lymphopenia [48]. IL-15 was also shown to impact the composition of the secondary T_MEM_ pool in response to infections [66]. The deficiency in the T_MEM_ pool is further corroborated using IL-2Rβ deficient mice where deficiency in IL-2 and IL-15 signalling promoted lineage decision towards secondary effectors by induction of T-bet and *Prdm1* (Blimp-1) over long-term memory cell promoting transcription factors Eomes, Bcl6 and Klf2 [67]. The differentiation of IEL in the intestine has also been shown to be dependent on IL-15-mediated regulation of T-bet expression. Tbx21^−/−^ thymic IEL precursors failed to expand and did not upregulate CD8αα when stimulated with IL-15 [68]. During ILC differentiation, IL-15 promotes the induction of Granzyme B in T cell/ILC progenitors, which cleaves NOTCH and prematurely switches off the differentiation towards T cell committed precursors [69]. Lastly, another essential homeostatic function of IL-15 is promoting the trafficking of memory CD8 T cells to tissues to establish tissue residency (T_RM_) at mucosal sites [70, 71]. However, the molecular mechanism regulating this during homeostasis is unclear.

### IL-15 signalling in inflammation and autoimmunity

Although IL-15 is an essential homeostatic cytokine, it also plays a key role in triggering effector T-cell responses in infection and sterile inflammation [1]. During bacterial or viral infections, Toll-like receptor or Type I Interferon mediated signals upregulate the expression of IL-15 in both antigen-presenting cells and epithelial cells. In autoimmune diseases, however, IL-15 appears to be induced in the absence of infection, and it is not clear what signals drive this upregulation. Small nucleotide polymorphisms (SNPs) in *IL15* and *IL15RA* have been shown to increase susceptibility to certain autoimmune diseases [72]. IL-15 has been implicated in the pathogenesis of various autoimmune conditions such as coeliac disease (CD), type 1 diabetes, rheumatoid arthritis (RA), psoriasis, multiple sclerosis (MS), vitiligo, systemic lupus erythematosus (SLE), and inflammatory bowel diseases (IBD). Emerging studies suggest that the chronic upregulation of IL-15 is the driving force behind the damage seen in some of these diseases, with a strong correlation reported between IL-15 levels and disease severity [73–76]. These observations suggest a crucial role of IL-15 in developing and progressing autoimmune conditions through mechanisms discussed below in detail (Fig. 3).

#### Lymphocyte survival in inflammation

As previously described, IL-15 is paramount for the homeostasis of tissue-resident cytotoxic lymphoid lineages. However, sustained, and excessive presence of survival signals can result in dysregulated immune cell expansion of the cytotoxic T cells, thus causing tissue damage [77–79]. Several studies suggest that constitutively enhanced expression of Bcl2 in lymphocytes can promote disease pathogenesis in animal models [80, 81]. Lymphocytes from SLE patients have a higher content of Bcl2 that can increase further upon incubation with IL-15 [82]. In refractory CD (RCD), Bcl-xl was shown to be the significant player promoting the survival of cytotoxic IEL [58]. Studies in refractory coeliac disease type II (RCDII) derived IEL lines treated with IL-15 showed that JAK3/STAT5 played a vital role in mediating survival advantage through the upregulation of Bcl-xl which could be by transcriptional activation of the promoter by direct binding of STAT5 [58]. In addition to Bcl2 family-mediated survival, IL-15-induced PI3K/AKT mediates deubiquitylation and stabilization of XBP1, which protects NK cells from stress-induced death. Unlike Bcl2, which is present at homeostasis, XBP1 is induced upon IL-15-mediated activation, suggesting a role in sustaining inflammatory conditions [83]. However, treatment of IL-15-activated NK cells with PI3K/AKT/mTOR inhibitors, RCDII IELs treated with AKT inhibitors did not show reduce viability compared to controls suggesting that this pathway might be dispensable for enhanced survival of cytotoxic lineages [58, 84].

#### Proliferation in inflammation

Compared to other common γc cytokines, increasing concentrations of IL-15 significantly enhanced the antigen-independent proliferation of human and murine NK cells, IEL, and T cells [85–87]. In SLE, IL-15 promoted the expansion of novel cytotoxic CD4^+^ CD28^−^ T cells, mainly driven by the JAK-STAT and PI3K-AKT pathways [88]. Patients with CD harbouring activating JAK1 (G1097) and STAT3 (D661) mutations have increased IL-15-mediated expansion of intestinal IEL. Similarly, phosphorylation of STAT3 in response to IL-15 was increased in refractory CD (RCD)-derived IEL lines with G1097D/C JAK1 mutations, and abolished by a JAK1 inhibitor, reducing their proliferation [69]. Treatment with JAK3 and STAT5 inhibitors completely abrogated proliferation in NK cells, suggesting a vital role for this pathway in proliferation [84]. While the PI3K/AKT pathway is dispensable for survival, studies in NK cells suggest it is essential for IL-15-induced proliferation. Treatment with AKT inhibitor (CAS 612847-09-3) or rapamycin (mTOR inhibitor) severely affected NK cell proliferation [84]. In RCDII-derived cell lines, treatment with PI3K inhibitor LY294002 also strongly inhibited proliferation triggered by IL-15 [58]. One of the mechanisms by which JAK/STAT and PI3K/AKT pathways promote proliferation during IL-15 signalling is through the upregulation of telomerase which maintains telomere length for continuous proliferation in NK, NKT, and T_MEM_ [89–92].

In addition, IL-15 stimulation also induces transcriptional programs that favour cell cycle entry as seen in T_MEM_ and IEL [87, 90]. Proteomic analysis of IEL stimulated with high levels of IL-15, to mimic the inflammation, found the induction of cell-cycle regulators, including the cyclin-dependent kinases, driving IEL entry into the S phase of the cell cycle [87]. High levels of IL-15 also induced the expression of the Proviral Integration of Moloney virus (PIM) family of kinases; PIM1 and PIM2 in IEL. In the absence of PIM1/2, IL-15-induced proliferation of IEL was completely blocked. Interestingly PIM proteins are not detectable with homeostatic IL-15 signals, but are strongly expressed with inflammatory levels of IL-15. PIM1 overexpression was also observed in IELs from CD biopsies suggesting conserved mechanism of IL-15-induced proliferation across species [87]. In addition, IL-15-mediated T-cell proliferation was shown to be also regulated through FK506-binding protein 12 (FKBP12) activation which in turn induces activation of p70-S6 kinase and ERK. Deficiency of FKBP12 led to decreased proliferation of T cells in response to IL-15. Along these lines, it was found that T cells from SLE patients had enhanced expression of FKBP12 [92, 93].

#### Tissue-residency and cell trafficking in inflammation

IL-15 signals are essential for driving tissue migration and residency of immune cells in homeostasis, but excessive infiltration of immune cells is associated with the pathogenesis of many diseases. IL-15 is an essential environmental cue required for the formation of tissue-resident memory cells by inducing Gcnt1 that upregulates glycosyl transferase enzymes required for the synthesis of ligands for P and E- selectins which facilitate extravasation into nonlymphoid tissues [ [94, 95]. In addition, IL-15 causes upregulation of CD69 expression which blocks sphingosine receptor SIPR1 surface expression that is known to promote exit into S1P-rich lymphatic circulation [96]. Transgenic mice overexpressing IL-15 had increased infiltration of CD8 T cells expressing NKG2D receptors in the intestinal tissue resulting in intestinal villous atrophy. Treatment with antibodies against IL-15 receptor reversed this affect [42]. Reduced CD8 T cells in intestine on IL-15 blockage was specific to IEC trans-presentation of IL-15 as epithelial-specific overexpression of IL-15 reinstated γδ IEL in the epithelial layer even with high IL-15 production in lamina propria [97]. In addition to tissue residency, IL-15 has been linked to promoting chemokinesis of T cells and inhibition of JAK1/3, STAT5, or PI3K signalling reduced IL-15-induced motility [97]. In this context, administration of IL-15 led to an increase in infiltrating CD8 T cells in mouse models of MS and RA and was associated with worse clinical outcomes [98, 99]. However, the mechanisms driven by IL-15 that promote tissue infiltration and residency during inflammation still need further exploration.

#### Cytotoxicity in inflammation

Enhanced cytotoxicity is one of the main factors responsible for disease severity during pathogenic infections and autoimmunity. The three IL-15-stimulated pathways drive various effector activities, such as proinflammatory cytokine synthesis, granzyme expression, and cytolytic activities, which are cell-type dependent. However, the signalling mechanisms driving the expression of effector proteins must be better characterized. IL-15 increased inflammatory capacity of NK cells by upregulating cytotoxic proteins such as granzymes, perforins, TRAIL, and NKG2D [84]. Enhanced cytotoxicity in NK cells is mediated by IL-15-induced AKT and ERK activation and blockade of the upstream molecules PI3K/MEK impaired cytokine production and degranulation [100–102].

Similarly, high levels of IL-15 can induce antigen-independent proinflammatory cytokine production and cytotoxicity in T cells through JAK3/STAT5 pathway [88, 102, 103]. Clinically, there is a strong correlation among IL-15 levels, IFNg, and cytotoxic capacity of IEL in CD and CD49a^+^ tissue-resident cells in psoriasis and vitiligo [73, 104]. IL-15 promotes the expression of various NK receptors, including CD94 and NKG2D, primarily in human TCRαβ CD8αβ IEL which drive antigen-independent cytotoxicity of these cells [105, 106]. IL-15-induced cytosolic phospholipase A_2_ (cPLA_2_) and arachidonic acid release in human intestinal cytotoxic T cells, which enhanced NKG2D-mediated killing by these cells [107]. Recently, it was shown that Granzyme B expression in murine IEL is mediated by IL-15-induced PIM kinases [87].

#### Metabolic rewiring during inflammation

Lymphocytes need to remodel their metabolism to cater to the increased energy demands of proliferation and effector functions. Hence, IL-15 signals also remodel the metabolism of effector cells during inflammation. IL-15 directly increases mitochondrial biogenesis, thereby enhancing spare respiratory capacity in T_MEM_. IL-15 also enhanced the expression of the rate-limiting enzyme of fatty acid oxidation, mitochondrial protein carnitine palmitoyltransferase 1A, which transfers fatty acids into the mitochondria for use in the TCA cycle [108]. IL-15 promoted the expression of antioxidant molecules in human CD8^+^ T cells such as glutathione reductase, thioredoxin reductase 1, peroxiredoxin, and superoxide dismutase, which are associated with increased survival and cytolytic activity [109]. In murine IEL, IL-15-induced ribosome biogenesis and protein synthesis, increased glucose uptake, and improved mitochondrial activity [87]. This metabolic reprogramming could be through the PI3K-mTOR pathway. In active CD duodenal biopsies, mTOR activity was higher compared to healthy intestines [110]. IL-15 also upregulates mTORC1 in NK cells, enhancing the metabolic activity of these cells (increased glycolysis and OXPHOS) and IL-15-mediated mTOR activation is necessary for GzmB expression and degranulation [101]. However, the exact signalling driving IL-15-mediated metabolic reprogramming in different cell types and their functions requires further clarification.

### Negative regulators of IL-15 signalling

All the studies on IL-15 signalling show that all three signalling pathways (JAK-STAT, PI3K/AKT, and MAPK pathways) play a role in the inflammatory response. As these pathways are also critical for maintaining homeostasis, the signal strength and persistence likely decide if the response will be inflammatory or homeostatic. The strength of the signal is also regulated by various negative regulators, which have also been shown to be dysregulated in pathogenesis.

Strong IL-15 signalling induces regulatory mechanisms that provide a negative feedback loop at multiple levels of IL-15 signalling. Members of the Suppressor of cytokine signalling (SOCS) family inhibit the enzymatic activity of JAKs by direct binding or by interacting with phospho-tyrosine residues via their central SH2 binding domain (Fig. 3). SOCS proteins recruit the ubiquitin transferase system with SOX box binding motif leading to target-directed proteasomal degradation [111]. SOCS1^−/−^ mice have a more significant proportion of autoreactive CD8 T cells that are hypersensitive to γc cytokines [111, 112]. Another member of the SOCS family, cytokine-inducible SH2-containing protein (CIS), was shown to be rapidly upregulated following IL-15 stimulation in NK cells and critically downmodulated JAK1 activity, such that CIS-deficient NK cells were hypersensitive to IL-15 [113]. Protein tyrosine phosphatase (PTP) containing SH2 domain, SHP1, also negatively regulates the JAK/STAT pathway. SHP1-deficient NKT cells from murine thymus and spleen showed enhanced proliferation when treated with IL-15, IL-2, or IL-7 [114]. Recently, thymocyte-expressed molecule involved in selection (Themis) was shown to be crucial for activating JAK/STAT in T_MEM_ stimulated with IL-15 [115]. Themis-deficient T_MEM_ stimulated with IL-15 had diminished JAK1, JAK3, and STAT5 phosphorylation compared to controls. Phosphorylation of the IL-15 signalling components was rescued by SHP1 phosphatase deficiency, indicating that Themis functions to release SHP1 inhibition of JAK/STAT signalling-induced by IL-15. IL-15 also induces lipid phosphatases SHIP1 and SHIP2, which are significant regulators of PI3K pathway [36]. Tumor necrosis factor-α (TNF-α)–induced protein-8 like-2 (TIPE2) was recently discovered to be a negative regulator of IL-15 triggered mTOR activity in NK cells [116]. Overexpression of TIPE2 in NK92 cells resulted in decreased phosphorylation of S6, a substrate of mTOR, and deficiency of TIPE2 increased phosphorylation of S6. Another PI3K-AKT pathway regulator is the deubiquitinase Otub1, identified as a negative regulator of IL-15 mediated priming of CD8 T and NK cells in mice [117]. Otub1 is recruited to the membrane upon IL-15 signalling, and deubiquitinates the PH binding domain of AKT, thus reducing its ability to bind to PIP_3_ and get activated. Deficiencies in these regulators have been associated with lack of tolerance to self, enhanced antitumor immunity, and hyperresponsive state of NK and CD8 T cells to IL-15 [111, 112, 116, 117]. These negative regulators ensure that the signalling proceeds in a controlled manner and thus prevent tissue damage.

## Conclusion and future perspectives

IL-15 is a master regulator of homeostatic as well as inflammatory responses (Fig. 4). Owing to the role of IL-15 in different pathologies, it is imperative to understand the differences in signalling under inflammation and at a steady state. Targeting inflammatory and cytotoxicity-specific signalling of IL-15 might be a useful strategy to treat autoimmune diseases. A recent review described the various therapeutic approaches developed to target IL-15 in autoimmunity, mainly by blocking its binding to its receptor [118]. IL-15 blocking antibodies have been successfully shown to reverse damage in mouse models of IL-15 mediated autoimmune diseases and several blocking antibodies have been progressed to clinical trial with varying degrees of success [72]. However, immune cell deficiencies such as loss of NK cells or CD8 + memory cells associated with blocking IL-15 are a concern, as the homeostatic as well as the inflammatory functions of IL-15 might be blocked by blocking receptor-ligand interactions. It has been shown that cis-presentation of IL-15 by heterotrimeric IL-15Rα/IL-2Rβ/γc receptor triggers fast and transient signalling whereas trans-presentation of IL-15/IL-15Rα to the heterodimeric IL-2Rβ/γc receptor-induced slow and persistent signalling [12]. In this context, one promising new strategy is to directly target the heterotrimeric IL-15Rα/IL-2Rβ/γc receptor that did not seem to affect homeostatic survival of NK and CD8 T cells, but targeted inflammation in tissues [119]. An Fc-coupled dimeric IL-15 mutein that selectively blocked cis-presentation of IL-15 without impacting trans-presentation was seen to specifically prevent inflammatory responses in mouse models of arthritis [119]. Although an increase in circulating IL-15 has been shown in several inflammatory conditions, it is unclear if the IL-15 is present as a complex with IL-15Rα. It would be interesting to understand if there is a shift in the IL-15 presentation mechanism as well as the expression of the heterotrimeric receptor to allow cis-presentation during tissue inflammation. While inflammatory IL-15 signals are mainly characterized by an increase in strength and signal duration, it may also be possible to target specific signalling proteins that are highly increased in expression or activity with continued stimulation of IL-15 signals in autoimmunity. Finally, IL-15 plays important roles in mucosal immunity to infections and in driving NK and T cell effector responses in cancer, and thus enhancing these inflammatory responses may be a useful strategy in mucosal vaccination and cancer immunotherapy.

## Data Availability

No data were presented in this review.

## Abbreviations

AKT/PKB AKT serine/threonine kinase 1: protein kinase B
CD: coeliac disease
cPLA_2_: cytosolic phospholipase A_2_
DETC: dendritic epidermal γδ T cells
FAM59A: fat facets in mouse 59A
GAB2: GRB2-associated binding protein 2
GRB2: growth factor receptor-bound protein2
IBD: inflammatory bowel diseases
IEL: intestinal intraepithelial T lymphocytes
IL: interleukin;
ILCs: innate-like lymphoid cells
LSP: long signal peptide
MAPK: mitogen-activated protein kinase
MS: multiple sclerosis
mTORC1: mTOR complex 1
NK: natural killer
NKT: NK like T cells
PDK1: phosphoinositide-dependent kinase 1
PERK: PKR-like ER kinase
PI3K: phosphatidylinositol-3kinases
PIM: proviral integration of moloney virus
PTP: protein tyrosine phosphatase
PTPN11: protein phosphatase non-receptor type 11
pY: phospho-tyrosine
RA: rheumatoid arthritis
RCD: refractory CD
RCDII: refractory coeliac disease type II
SH2: src-homology 2
SLE: systemic lupus erythematosus
SNPs: ssmallnucleotide polymorphisms
SOCS: ssuppressorof cytokine signalling
SP: signal peptides
SSP: short signal peptide
STAT: signal transducers and activator of transcription
TMEM: CD8^+^ memory T
T_reg_: regulatory T cells
T_RM_: tissue resident memory T cells
γc: : γ-chain
